# Quality assurance in transnational higher education: a case study of the tropEd network

**DOI:** 10.1186/1472-6920-13-43

**Published:** 2013-03-27

**Authors:** Prisca A C Zwanikken, Bernadette Peterhans, Lorraine Dardis, Albert Scherpbier

**Affiliations:** 1 Royal Tropical Institute, Amsterdam, the Netherlands; 2 Swiss Tropical and Public Health Institute, Basel, Switzerland; 3 Office for International Affairs, University College London, London, United Kingdom; 4 Faculty of Health, Medicine and Life Sciences, Maastricht University, Maastricht, the Netherlands

**Keywords:** Quality assurance, Higher education, Cross-border, Transnational, Networks

## Abstract

**Introduction:**

Transnational or cross-border higher education has rapidly expanded since the 1980s. Together with that expansion issues on quality assurance came to the forefront. This article aims to identify key issues regarding quality assurance of transnational higher education and discusses the quality assurance of the tropEd Network for International Health in Higher Education in relation to these key issues.

**Methods:**

Literature review and review of documents.

**Results:**

From the literature the following key issues regarding transnational quality assurance were identified and explored: comparability of quality assurance frameworks, true collaboration versus erosion of national education sovereignty, accreditation agencies and transparency. The tropEd network developed a transnational quality assurance framework for the network. The network accredits modules through a rigorous process which has been accepted by major stakeholders. This process was a participatory learning process and at the same time the process worked positive for the relations between the institutions.

**Discussion:**

The development of the quality assurance framework and the process provides a potential example for others.

## Introduction

Transnational or cross-border higher education has rapidly expanded since the eighties [1]. Since the mid nineties higher education (HE) has fallen under the framework of the World Trade Organisation (WTO) and General Agreement on Trade in Services (GATS) [2]. In this context, transnational education has become a commodity, increasing the international trade of educational services [3]. This expansion was mainly economically inspired, aiming to boost revenue, but was also driven by aims relating to capacity building, developing human resources, identifying talented students to work in the host country, and increasing international understanding [2-6]. Due to the rapid rise in transnational education by profit and non-profit providers, as well as the commodification of HE [7,8] issues regarding quality assurance came to the forefront [1,2]. In Europe the Bologna process spurred interest in quality assurance as well as the transfer of credits [9,10]. With the development of double and joint degrees, reluctance in recognizing education followed at other universities had to be overcome [11], while other quality assurance issues remained [12,13]. UNESCO (United Nations Educational and Scientific Organisation) developed guidelines regarding the quality of cross-border education. However, these guidelines are voluntary [14]. The World Federation for Medical Education (WFME) formulated guidelines for accreditation of postgraduate medical education as well as global standards for quality improvement of medical education [15]. However, many issues regarding transnational HE remain unresolved, particularly regarding quality assurance.

### Definitions

According to UNESCO 2005 the definition of cross-border HE is: “includes higher education where teacher, student, program, institutions/ provider or course materials cross national jurisdictional borders”. Cross-border higher education and transnational higher education are often used interchangeably [2]. Therefore both terms will be used interchangeably in the article.

The definition of quality assurance in HE has evolved in the last ten years. Woodhouse [16] referred to quality assurance as relating “to the policies, attitudes, actions and procedures necessary to ensure that quality is being maintained and enhanced”. According to Harvey [17] after much discussion and input: “Assurance of quality in higher education is a process of establishing stakeholder confidence that provision (input, process and outcomes) fulfils expectations or measures up to threshold minimum requirements”. According to UNESCO (2005) the following stakeholders in higher education can be distinguished: governments; higher education institutions/providers including academic staff; student bodies; quality assurance and accreditation bodies; academic recognition and professional bodies [14].

Schüle (2006) defines a double and joint degree as follows, using as a basis the definition provided by the European Commission (latest update 2009): a “double degree: two nationally-recognised diplomas issued separately by the universities involved in the integrated study programme”, and a “Joint degree: a single diploma issued by two or more institutions offering an integrated study programme. The single diploma (Bachelor, Master, Doctor) is signed by the rectors of all participating universities and recognised as substitute of the national diplomas” [11,18].

### Background of the tropEd network

The initiative to create a higher education network in international health came from the directors of public/tropical health institutions who collaborated in Tropmedeurop, an association centred on tropical medicine education in Europe, in 1994. A new formalized network focused on education was established in 1996, initially with 13 institutions in Europe, named TropEdEurop. Now renamed, tropEd, the network includes more than 30 institutions of higher education in international and global health in Europe, Africa, Asia, Australia and Latin America. The tropEd network includes almost all institutions in Western Europe offering a MIH and a range of institutions outside Europe offering modules, the word largest network for a Master in International Health (http://www.troped.org). The tropEd General Assembly (GA) of the network meets three times a year. Full members in the network are institutions of higher education recognized by a national authority. The voting members are 1 representative of each institution who is a full member; voting results are by majority.

**Figure 1:**
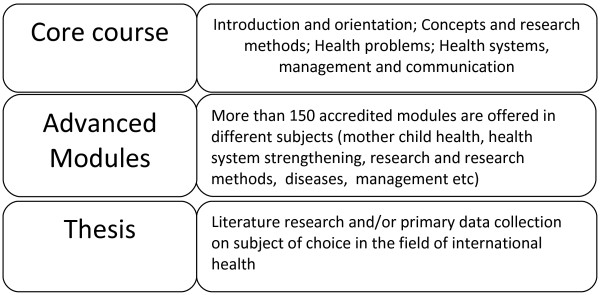
Schematic view of a tropEd master in international health program.

The network has developed a robust common framework for a Masters in International Health (MIH), see Figure 1. However, the network does not deliver the degree directly; rather, the member institutions do. The framework defines common minimum academic and quality assurance structures, content and criteria to which the nationally accredited degree must adhere in order to be recognized as a ‘tropEd MIH’. This recognition is framed around a Masters level program of 60 European Credits (EC) of the European Credit Transfer system (ECTS). An institution is categorized as the ‘home institution’, if a student can enter the Masters program there and complete a ‘core course’ of 3 months, equivalent to 20 EC. In 2011, 8 institutions classified as home institutions. The home institution also provides tutorial support to the student throughout the program and awards the final Masters degree. TropEd students are expected to acquire up to 10 ECTS through advanced modules at a tropEd institution outside the country of their home institution depending on institutional regulations.

In addition to the degree from their home institution, graduated Masters students receive tropEd recognition if they complied with the following criteria in addition to the mobility requirement: at least two years of relevant professional experience of which at least one year in low- and middle-income countries/societies, Master degree obtained from home institution and completion of the masters program within 5 years. In 2010, there were 479 students from different home institutions registered in the tropEd network.

Given the number of partners involved in the development and functioning of the educational network, the diversity of national structures, traditions and educational practices, there has been an consistent need for quality assurance to generate and maintain high quality standards of education.

This article aims to identify key issues regarding quality assurance of transnational higher education and discusses the quality assurance of the tropEd Network for International Health in Higher Education in relation to these key issues.

## Methods

The methods used were literature review and review of documents of tropEd.

For the literature review the search terms were: higher education AND (international or transnational or cross-border) AND quality assurance or quality improvement or quality control or accreditation or quality standards or joint degrees or double degrees or “international accreditation”. The databases searched were Pubmed, Google Scholar and Scopus. The Search period was 1990 – 2011, the search date: 13 July 2011. Keywords were searched in title/abstract/keywords; the language was limited to English. This led to 94 unique titles, of which 41 were considered relevant by the first three authors. Articles discussing distance higher education, quality assurance of professions, quality improvement, total quality management or focusing on one country only were excluded. The first author reviewed all 41 articles; the second and third author read each half of the articles (ie 20 and 21 articles). The articles were discussed until four major themes emerged as mentioned below.

The research team reviewed the following documents of tropEd: all GA meeting minutes, from 2005–2011, all minutes of the Quality Assurance Committee (from 2005–2011), all minutes of the troped Erasmus Mundus consortium (2005–2011), statutes, profile, strategic plan, work plan, annual plans, procedures and guidelines, for example: guidelines for core course and optional/ advanced modules, ethical guidelines, handbook for core course/ advanced module accreditation, professional profile, institutional self-evaluation form, and forms for tropEd recognition as well as thesis guidelines using a checklist and a topic list. The checklist centered on the accreditation of the advanced module and core courses ie numbers and year. The topic list included topics such as development process of criteria, institutional agenda, transparency, decision making.

## Findings

### Issues regarding quality assurance in cross-border or transnational higher education

Almost all relevant articles found in the literature search dealt with issues of quality assurance internationally relating to when a higher education institution ventured towards other countries either through branch campuses, franchising courses or working with partners to jointly deliver educational programs. In reported cases, most featured the USA, Great Britain or Australia [4,19-22]. However, no description of the quality assurance process and results of a higher postgraduate education network was found.

Key themes regarding quality assurance of cross-border education which emerged from the literature review and the tropEd experience are explored below: true collaboration versus erosion of national education sovereignty, equivalence and comparability of quality assurance frameworks, accreditation agencies and transparency.

1. True collaboration versus erosion of national education sovereignty

1.1. Literature

In the literature Hodson argues that the then existing collaborative audit approaches in ‘overseas’ higher education lacked cultural sensitivity [4]. Both Gift and Smith concur and argue from an exporting- importing model that a true collaboration between universities is often blurred by the quality demands of the exporting country or institution [21,23]. Smith, analyzing the codes of practice of three major HE exporters (UK, USA and Australia), reveals that some codes leave little room for adaptation to ‘cultural mores’ [21]. Some authors view these developments with caution, arguing that countries which are at the receiving end may have difficulties safeguarding the relevance of the education, their culture and their educational sovereignty [3,23], with HE even becoming elitist [8]. Stella discusses how cross-border HE often disadvantages developing countries as they are unable to participate effectively in the global trading system [2]. Gu, however, also argued that continental European countries tend to act on regional integration and complement each other with their strengths [3].

1.2. tropEd

In the tropEd network the developed quality system was created through *participatory learning for all members*: from the beginning, the network decided not to have a separate curriculum committee, but rather to have every institution involved. The GA decided to aim for an open process, where the GA acted as the curriculum committee, so each member could read and comment on each core course and advanced module.

During the *process* of developing the quality assurance often a small group of interested representatives from different institutions worked together on different topics. This process was quite informal: during a discussion in the GA an issue needing elaboration would be identified, no terms of reference were made, and every interested GA member could join the discussion. The group would come together, sometimes during the GA meeting or sometimes in small-group meetings in between the GA meetings. Results of the discussions would be brought back and re-discussed in the GA. When necessary the small group would take the comments and suggestions from the GA and further revise before returning it to the GA for consensus. Through this process the network developed a number of documents and guidelines i.e. guidelines for core course and advanced modules, ethical guidelines, a strategic plan for the network, handbook and forms for tropEd recognition as well as thesis guidelines.

As in any multicultural and multi-country network with different interests, *resistance to change* or to proposed procedures sometimes emerged. Within the network this resistance was often addressed through informal discussions.

Sometimes resistance emerged due to *institutional agendas* i.e. difference in course fee or difference in institutional procedures. These agendas were clarified, often during the GA and informal discussion, and then issues were reviewed, and possible accommodation or help/ support was offered to the members on how to deal with the resistance within the institution.

To foster student mobility and with initial members being only European institutions, the network adopted the European Credit Transfer System (ECTS), given its explicit design to foster mobility within Europe. The institutions outside Europe who joined the network later kept their own credit system, while using the ECTS for tropEd students.

2. Equivalence and comparability of quality assurance frameworks

**Table 1:** Nr of courses reviewed and outcome in the tropEd network from 2007-2011

**Nr of advanced modules per year/ outcome**	**Total reviewed**	**Accepted at once**	**Resubmission with minor changes**	**Resubmission with major changes**	**Rejected**
2007	46	11	10	25	-
2008	67	24	19	24	-
2009	48	17	15	15	1
2010	59	11	19	27	2
2011	49	8	21	20	-
Total advanced modules	269	71 (27%)	84 (31%)	111 (41%)	3 (1%)

Source: minutes of the tropEd General Assembly meeting from: 2007–2011.

2.1. Literature

According to literature from the nineties onwards, increasing international mobility, and therefore international comparability, became an important issue, especially in Europe and the USA [21,24]. Quality assurance was very often discussed from the viewpoint of ‘provider’ and ’receiver’ institutions and countries: the degree of autonomy of either branches or local institutions granted by the ‘home’ institution to adhere to procedures of the ’home’ institution or develop their own quality assurance processes [19-21,25]. Stella states that national frameworks for quality assurance of cross-border education are not well developed [2], though Murray argues that for Australia a sophisticated framework for monitoring of cross-border higher education exists [26]. Bolton argues that existing quality assurance frameworks often do not allow accommodation of manageable risks associated with innovation, flexibility and experimentation in new market places, discussing a partnership between Australia and China [27]. Billing sees especially in Europe a ‘general’ model of quality assurance developing [28].

2.2. tropEd

In the tropEd network to admit a new *institution* the GA developed a standardized procedure, thereby checking its quality: the new institution has to subscribe to the definition of International Health by tropEd, complete a self evaluation and undergo an institutional site visit. The GA which meets three times a year, decides on official admission to the network through deliberation and voting. The GA developed a guideline for the self evaluation which includes details regarding i.e. academic background, faculty, services, research and resources and the site visit, which details i.e. discussion with students, staff, management and an ocular survey of the teaching and learning facilities. During the last five years (2007–2011) 18 institutions applied to become a member, 13 institutions were visited during a site visit, 10 of those institutions became a member of the network (tropEd GA meeting minutes 2007–2011). One became a member in 2012, other institutions’ membership is still pending or they declined.

To develop the Masters program at the start of the network in 1996 the GA defined standards for the core course, advanced modules and thesis. In 2004 the GA developed a professional profile of the graduate MIH, including professional competencies and overall learning objectives.

For the core course and the advanced modules the GA developed quality criteria. Later the criteria for the assessment of the core course and advanced modules became more refined, including title, learning objectives, content and alignment of assessment with learning objectives. Subsequently, further criteria were developed to support more in-depth learning, i.e. aligning learning methods with learning objectives and assessment. The curriculum content was checked for appropriateness to Masters level.

The guidelines for the core course and the advanced modules were *binding*: if an institute submitted a core course or module which the GA did not accept, the core course or module would be rejected and the representative needed to go back to the institution to re-discuss the core course or module for resubmission. In the minutes of the GA the written explanation was provided as to why the core course or advanced module was rejected. The GA needs to review and accredit the core course and advanced modules every five years. According to tropEd regulations the Executive Committee approves the core course or advanced modules with minor changes upon resubmission. The GA needs to approve major changes.

From 2007–2011 the GA reviewed 8 core courses of the 8 home institutions; 2 were accepted at once, 4 had to be resubmitted with minor changes and 2 to be resubmitted with major changes, see Table 1.

From 2007–2011 the GA reviewed 269 advanced modules, of which 71 were accepted at once. Out of the 195 rejected advanced modules, 84 modules had to be resubmitted with minor changes. The other 111 modules required major changes. Three were rejected, meaning that they were not suitable for the MIH, see Table 1. The accreditation process was quite rigorous, as the GA accepted only 25% of the core courses and only 27% of the advanced modules at once, see Table 1.

For the thesis, as guidelines per institution differed, the GA developed generic thesis requirements, including ethical guidelines, which could be adapted by each institution.

In 2004 8 tropEd member institutions established a consortium to offer 5 fulltime MIH study tracks MIH financially supported through the Erasmus Mundus program by the European Commission. Initially 1 track, and later 3 tracks offered joint degree awards while institutions of the other tracks offered double degree awards. The institutions worked closely together to align the study programs, to secure proper hand-over of students from one institution to the next institution and to fulfill all the administrative requirements to offer the double or joint degrees. Discussions and decisions regarding the Erasmus Mundus program were always reported in the GA. Because of the double and joint degree programs the participating institutions developed joint selection criteria for the scholarships. Except for the administrative issues the double and joint degree programs did not have implications for the quality assurance of the network as a whole. Because the network had an elaborate quality assurance system the tropEd Erasmus Mundus consortium was easily formed with the 8 institutions that choose to join.

3. Accreditation agencies

3.1. Literature

The literature poses a range of challenges regarding recognition of higher education institutions and courses across borders. The rapid increase of HE institutions which are not accredited in their home country, nor in the country in which they offer their cross-border education, leads to questions regarding the capability and credibility of national and international accreditation agencies [1].

Concerns about the quality, consistency and relevance of accreditation are reflected internationally. Gu argues that in China there is insufficient knowledge and experience in quality assurance of transnational education, as most existing systems of quality assurance and accreditation focus on the local higher education system [3]. The case of Malaysia, an export hub of HE, demonstrates the challenge of getting national accreditation accepted internationally [29]. A case study of Kenya revealed that one foreign provider was locally accredited, yet other cross-border providers or education offered were not accredited through their home country nor geared towards the needs of the country [30]. Knight [31] warns of accreditation mills in the context of cross-border education. In Taiwan the discussion centers on the quality and national accreditation of international accreditation agencies, plus the additional administrative burden [22]. With the emerging trend of institutions seeking accreditation internationally, increasing administrative burdens as well as possibly conflicts may arise due to the different requirements by the different agencies, thereby decreasing efficiency [22,24,32].

A range of regional and global responses and frameworks have attempted to address such challenges. In the Caribbean, the withdrawal of the British accreditation and a rapid increase of foreign providers of HE, led to the Caribbean Community and Common Market (CARICOM) establishing a regional mechanism for accreditation, to guide governments in developing national mechanisms [23]. In Latin America, six countries joined forces in MERCOSUR to recognize each other’s accreditation for certain degrees, provided auditors from other countries had collaborated in the accreditation [33]. Haug (2003) suggests a meta-accreditation mechanism for Europe i.e. an accreditation of the accreditation agencies, thereby reducing costs, which nationally can be quite high [34]. In Europe, with the establishment of the European Network of Quality Assurance Agencies (ENQA) as part of the Bologna process and the increasing tendency of national accreditation agencies to recognize each other’s accreditation, there seems to be some progress on a number of issues. Stella urges increasing cooperation among quality assurance agencies in furthering the UNESCO guidelines on quality assurance of cross-border education [2]. Van Damme advocates a global regulatory framework [35]. The WFME developed accreditation standards for postgraduate medical education to stimulate local development of standards and to facilitate the acceptance of doctors in countries other than where they were trained [36].

3.2. tropEd

tropEd has developed an internal framework to act as its own ‘accrediting’ agent due to the lack of accreditation bodies for transnational higher education at international or global level. Universities, in countries where they hold degree-awarding powers, as well as national accreditation bodies, have accepted the tropEd accreditation of programmes as well as advanced modules followed by students in member institutions in other countries. The acceptance of tropEd accreditation by these national accreditation bodies can be seen as a benchmark for tropEd.

4. Transparency

4.1. Literature

In the literature Machado argues that the rapid increase of new providers demands greater clarification and transparency regarding the normative basis of transnational education [1]. National governments should regulate i.e. protect educational titles, and the public should be informed. Machado sees a critical role for the ENQA in Europe, Knight discusses the role of UNESCO and the regional conventions [1,31]. Shanley argues for the use of a website in increasing transparency in a network of undergraduate education across Europe [37]. Additionally, Bolton in her article on a Chinese-Australian collaborative educational alliance argues that transparency towards stakeholders is important to create value of the degree [27].

4.2. tropEd

Within the tropEd network transparency was enhanced by involving and learning from the students. By keeping in close contact with the students through involving an elected tropEd student representative in the GA meetings, the network has been able to respond to feedback on issues of importance to students. This openness meant that issues were voiced and could be taken up at a very early stage, so that the network or member institution would be able to implement improvements. Quality criteria have been revised and refined in an open process involving the full GA in decision making.

When the core course or advanced modules are submitted for reaccreditation, institutions have to give a summary of the evaluations by students over recent years, this is also published on the internet. On the internet students can find when a course or module was accredited and until when the accreditation is valid.

In the tropEd network the degree is issued in a specific country, meaning that each institution needs to ensure that the course followed outside the country and within the network is recognized. Up to now that has never been an issue, possibly due to the fact that individual institutions can show the rigorous quality assurance process applied by tropEd in accrediting the core course and advanced modules. Furthermore, currently there are no specific professional bodies accrediting degrees for MIH, perhaps owing to the multi-professional and multidisciplinary nature of International Health as well as the globalised context in which these graduates work.

## Discussion

Linking the specific experience of the tropEd network with the broader issues in the literature, a deeper understanding emerges on the key themes:

• True collaboration versus national education sovereignty

Member institutions have been able to collaborate in improving their quality through peer review as well as learning from each other in International Health. The network functions well although not always the same institutional representatives can attend meetings and despite the fact that members are to some extent competitors for the same potential students. Some members are only able to the Masters offer degree by including advanced modules from other institutions so there is a utilitarian aspect to some extent for these institutions. Other members can offer the entire degree on their own but want to be part of a bigger network due to the benefits for students, the organizational learning, as well as raising the profile of international health as an academic and professional field. Some members have been able to develop new advanced modules based on the learning within the network or developed modules together. Individual institutions felt that they benefitted being a member of the network; benefits cited are: the harmonization of contents, information for tutoring of students, validation of own standards and procedures, sharing global developments in international health for the content of courses, the development of new learning approaches, of quality improvement of own modules, of common understanding of quality standards in teaching and learning. Challenges mentioned are the frequency of the meetings, the timing of the meetings, complying with all the requirements for module accreditation and reaccreditation as well as agreeing within institutions to allow students to take modules at other institutions. As Gu argues that European countries complement each other [3], tropEd started as a European network of institutions complementing each other to offer a MIH. Institutions outside Europe who joined later, have been able to adhere to the quality assurance standards and contributing actively to the improvement.

• Equivalence and comparability of quality assurance frameworks

The tropEd network started with European institutions first, and was aided by the Bologna process, which provided a structure for harmonizing practice across Europe. In more recent years membership from outside of Europe has extended and deepened the reach of its shared quality assurance. As Billing saw a ‘general’ model of quality assurance developing in Europe, tropEd becoming increasingly global, is developing towards a global quality assurance model [28].

• Accreditation agencies

Because no accreditation agency for a worldwide transnational higher education network exists, tropEd ‘accredits’ its own core course and advanced modules. The acceptance by national accreditation agencies of the accreditation of tropEd of modules taken outside the country by students implies that they find the quality assurance framework of tropEd credible. However the additional burden of further quality assurance measures alongside national and institutional quality assurance requirements has been mentioned [22] and the costs of running the network remain a challenge. The recognition of the network outside the network itself, for example by the EU through the Erasmus Mundus scholarships (2004, 2009) indicates EC approval of the tropEd framework and quality assurance standards. The tropEd network was also mentioned as an example of best practice by Ecotec Research and Consulting Ltd, commissioned by the European Commission, Directorate General for Education and Culture when studying quality assurance across Erasmus Mundus consortia [38]. The question is whether tropEd would like to remain its own accreditation agency or that the ENQA or UNESCO could play a role.

• Transparency

The tropEd network has worked hard on improving its transparency towards potential students. Stakeholder engagement with regards to i.e. employers is under development. Professional bodies for MIH graduates do not exist yet. Whether achieving tropEd recognition by graduates improved the outcome has been subject of another study [39]. Because of the quality assurance procedures, institutions have recognized each others’ core course and advanced modules followed by the students, unlike experiences within the Erasmus program in Europe, where universities had difficulties recognizing each other’s credits [11].

## Limitations

For the study review of literature and review of documents were used. Observation and key informant interviews could have been conducted, however this was compensated by the fact that three of the authors were longstanding representatives of their respective institutions in the network with an in-depth knowledge of the development of the quality assurance and have participated either one, often two or three in all the GA meetings of the network. Though being a representative of their respective institution might constitute a bias, in-depth discussions amongst the research team during the writing of this paper deepened the understanding of the process of development.

Areas for further research could include the role of the student representative, the governance of the network, to define and study outcome and impact indicators, the impact of the tropEd quality assurance process on the actual improvement of quality of the education provided as well as student/ graduate performance or workplace/ employment success.

## Conclusion

The quality assurance process in the tropEd network was and still is a participatory learning process and requires time. Quality assurance within the network has been formalized but in such a way that it is fully integrated in the functioning and learning of the network. Members of the network feel ownership of the QA standards, documents and processes and have the ability to change them and develop them. However, this process requires respect, trust and sharing tasks among the different partners. Given that in transnational HE no quality assurance frameworks or accreditation exist, tropEd has constructed an evolved and shared quality assurance structure, the validity of which has been accepted by national and international agencies and could be an example for others.

## Abbreviations

HE:Higher education; MIH: Master of International Health; WFME: World Federation of Medical Education; UNESCO: United Nations Educational and Scientific Organisation.

## Competing interest

PZ, BP and LD have been members of the Executive Committee and the Quality Assurance Committee of tropEd, and PZ has been the chair of the Quality Assurance Committee of tropEd.

## Authors’ contribution

PZ initiated the article. All authors were involved in the conceptualization, PZ, BP and LD all contributed to reviewing the articles. All authors contributed to writing and review. All authors agreed with the final content.

## Authors’ information

PZ, MD, MScCH is a specialist in public health, human resource development, training and HIV/AIDS. She has extensive experience in developing and organizing degree and short courses in public health, including quality assurance as well as module development for e-learning. Currently, Dr Zwanikken is the Royal Tropical Institute’s Area leader for Education and program director of the MIH.

BP, nurse, MScPHDC is the Coordinator in-charge for a 3 months Diploma Course and advanced modules in health district management within the Master of International Health program and has extensive experience in international teaching/training networks and in assessment and evaluations of health systems in conflict /postconflict countries.

LD is the Senior Executive Officer (Partnerships and Collaborations) at University College London. Her quality assurance expertise includes more than a decade of working in international HE partnership networks, and coordinating an Erasmus Mundus joint Masters programme. She is currently completing a PhD at the Institute of Education in London, UK.

AS, MD, PhD is the dean of the Faculty of Health, Medicine and Life Sciences at Maastricht University. He is also professor of quality improvement in medical education and consultant in many international projects.

## Pre-publication history

The pre-publication history for this paper can be accessed here:

http://www.biomedcentral.com/1472-6920/13/43/prepub
